# Convince Yourself to Do the Right Thing: The Effects of Provided Versus Self-Generated Arguments on Rule Compliance and Perceived Importance of Socially Desirable Behavior

**DOI:** 10.3389/fpsyg.2020.613418

**Published:** 2020-12-23

**Authors:** Nieke Lemmen, Kees Keizer, Thijs Bouman, Linda Steg

**Affiliations:** Department of Psychology, Faculty of Behavioural and Social Sciences, University of Groningen, Groningen, Netherlands

**Keywords:** intrinsic motivation, intervention, arguments, rule compliance, self-persuasion

## Abstract

One way to enhance rule compliance is to provide people with arguments explaining why the desired behavior is important. We argue that there might be another, potentially more effective way to enhance rule compliance: ask people to generate arguments in favor of the rule themselves, which can trigger a process of self-persuasion. We compared the effects of providing arguments, asking respondents to generate arguments themselves, and a combination of both approaches on rule compliance and the perceived importance of the rule. A field experiment revealed that rule compliance was higher in all experimental conditions compared to a control condition, with the highest level of rule compliance in the conditions that either presented the arguments or asked people to generate arguments themselves. Yet the rule was only evaluated as more important compared to the control condition, when people generated arguments themselves. This study suggests that rule compliance and perceived importance of this rule can be enhanced by easy low-cost interventions.

## Introduction

Many organizations implement rules to encourage socially desired behavior. It is assumed that providing arguments explaining why it is important to follow such rules will increase rule compliance. For instance, prohibitions signs set up near lakes and forests explaining that littering is not allowed in order to conserve and protect the natural environment or persuasive messages placed along highways to convince drivers that it is important to keep to lowered speed limits in order to reduce accidents and emissions. Likewise, Primate Park Apenheul, a zoo in Netherlands where the current research was conducted, has implemented a rule that visitors are not allowed to lure monkeys in areas where they roam freely amongst visitors. To increase rule compliance, they provide visitors with arguments explaining the rationale behind this rule. Specifically, visitors are told not to lure the monkeys because this intimidates the monkeys and disturbs their natural behavior. The assumption is that such arguments will make it more likely that people acknowledge the importance of the rule and comply to it.

There is some evidence to suggest that providing arguments explaining the importance of a desired behavior can result in more positive attitudes toward this desired behavior (Wänke et al., 1996), particularly when the arguments are strong and provided by a relevant and credible source (Kaufman et al., 1999; Ziegler et al., 2005; Smith et al., 2013). However, it is not clear whether providing arguments will also make people find the behavior *more important*, and whether this will make them more likely to *engage* in the desired behavior. We address this gap in the current study. On the basis of the above, we hypothesize that visitors of Apenheul will be more likely to act in line with the non-luring rule (hypothesis 1a) and will find the non-luring rule more important (hypothesis 1b) when Apenheul, a source that can be considered credible and relevant when it comes to monkey well-being, provides them with arguments for why visitors should not lure the monkeys compared to when Apenheul does not provide these arguments.

Yet, we propose that there may be an even more effective way to increase the perceived importance of a rule and promote rule compliance: trigger people to come up with reasons for engaging in the desired behavior themselves, so that they convince themselves of the importance of the rule rather than being persuaded by an external source (cf. Aronson, 1999). Such a self-persuasion procedure is likely to motivate people to adopt the desired behavior, as they are motivated to be consistent and to act in line with their own arguments (Cialdini, 2001). After all, if you came up with arguments in favor of performing a certain behavior yourself, you apparently find this behavior important. This, in turn, may motivate you to act in line with the rule so that you will behave in accordance to your own arguments. There is some initial evidence to suggest that self-persuasion may be more effective than providing arguments. People who were asked to write down arguments why they should exercise more regularly had more positive attitudes toward exercising and a stronger intention to exercise more than people who were asked to read arguments why exercising is beneficial (e.g., Baldwin et al., 2013). We extend this research by studying the effects of providing arguments versus asking people to generate arguments themselves on the perceived importance of the rule and on actual behavior in accordance to the rule. We hypothesize that asking people to come up with their own arguments in favor of a rule will promote engagement in the desired behavior more (hypothesis 2a) and increase the perceived importance of the rule more (hypothesis 2b) than when arguments are provided by an external source and compared to not providing or asking for arguments.

Further, we will explore what would happen to the perceived importance and adherence to the non-luring rule if we combine the two interventions such that arguments are offered first before asking people to come up with their own arguments. Two competing hypotheses could be formulated in this regard. On the one hand combining both interventions could weaken the positive effect of self-persuasion because people may not feel they generated the arguments themselves, but rather that the arguments were provided by an external source. This would undermine the self-persuasion process, as the basic premise of self-persuasion is that the motivation to act comes from within the person (e.g., Aronson, 1999). In fact, combining both interventions may even result in reactance (Brehm and Brehm, 1981; Rains, 2013) and decrease the perceived importance and likelihood of adopting the desired behavior, if people feel that they are being checked upon to see if they understand and remember the arguments provided. On the other hand, combining both approaches could strengthen the self-persuasion effect by making it easier for people to come up with arguments themselves. Indeed, research suggests that the easier people find it to think of arguments in favor of a rule, the more likely it is that they will convince themselves of the importance of the rule and adopt the desired behavior (i.e., availability heuristic, Tversky and Kahneman, 1973; Wänke et al., 1996; Wänke and Bless, 2000). This reasoning implies that people will evaluate the rule as more important and that they will be more likely to adopt the desired behavior when both approaches are combined, as it will be easier for them to come up with arguments in favor of this rule.

We will investigate which of these two reasonings is most plausible by testing whether providing visitors with arguments in favor of the rule to not lure the monkeys, before asking them to come up with arguments themselves will increase or decrease the perceived importance of the non-luring rule and the likelihood of visitors adopting the desired behavior compared to either solely providing arguments or solely asking them to come up with arguments.

## Methods

To test our reasoning, we conducted a field study in collaboration with Primate Park Apenheul. The study took place in and around an area in the park in which small squirrel monkeys roam freely among the visitors. As a general rule of the park, visitors are not allowed to lure the monkeys^1^. In our study, visitors were exposed to one of four experimental conditions conveying a message to promote the non-luring rule (see below) right before they entered the free-ranging area. The study comprised of two parts. In study 1a, we observed the rule compliance of 2,264 visitors inside the free-ranging area. In study 1b, we asked 350 visitors that were seated at a terrace after the exit of the free-ranging area to fill out a paper-and-pencil questionnaire in which they answered questions about the importance of the non-luring rule. The study was approved by the Ethical Committee of Psychology of the University of Groningen.

### Design

**FIGURE 1:**
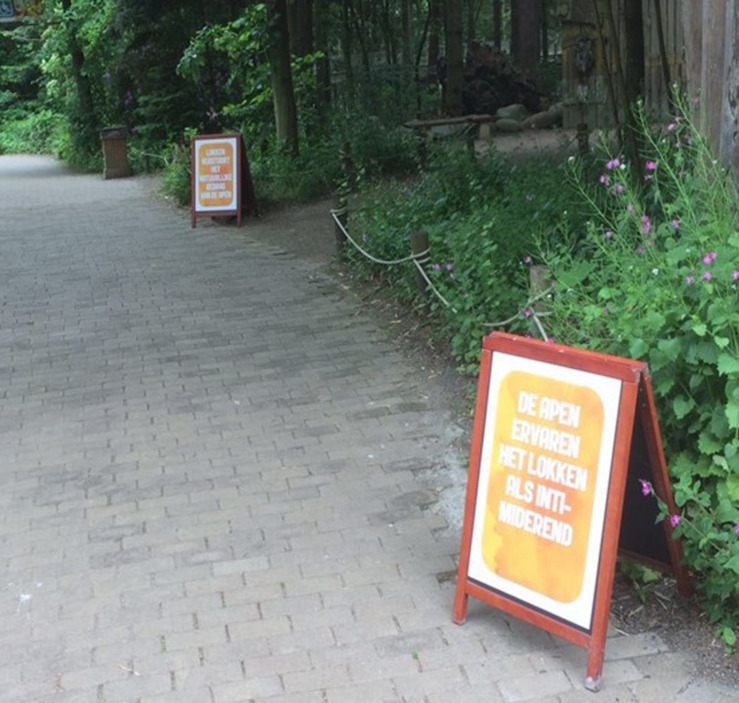
Signs with arguments in favor of the non-luring rule.

**FIGURE 2:**
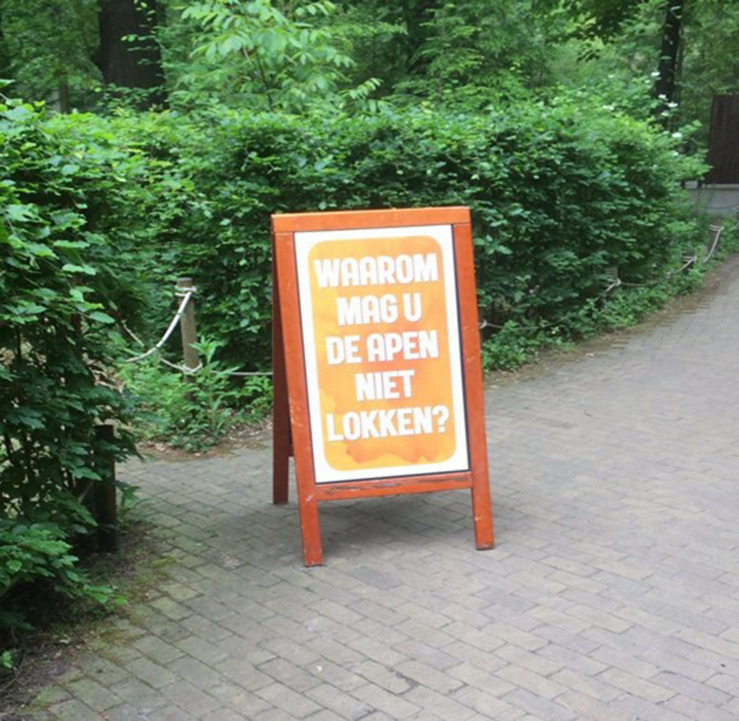
Sign with question: “why should you not lure the monkeys?”.

We followed a 2 (providing arguments yes/no) x 2 (asking question yes/no) between-subjects design. Specifically, we first systematically varied whether or not arguments in favor of the non-luring rule were presented. Arguments for why people should not lure the monkeys were presented on three signs (55 × 85 cm) that were placed right before the entrance of the free-ranging area. Each of the three signs displayed one of three arguments in favor of the non-luring rule: “luring intimidates the monkeys,” “luring disrupts the natural behavior of the monkeys” and “luring disrupts the strict rank that exists within the (monkey) group” (see Figure 1). The arguments were formulated after consulting (animal) experts of the Primate Park^2^. Second, we systematically varied whether or not the question “Why should you not lure the monkeys?” was presented to the visitors through one large (71.5 cm × 138.5 cm), noticeable sign right before the entrance of the free-ranging area (see Figure 2).

All signs used within the study were designed to fit with the style of the other signage in Apenheul. To increase the probability that participants read the signs, the signs were placed alongside a path surrounded by only trees, with no other stimuli or distracters.

Table 1 gives an overview of the four different conditions. We tested the effects of the four conditions on the percentage of visitors who violated the non-luring rule during the testing period (Study 1a) and the perceived importance of the rule (Study 1b). In all conditions, the non-luring rule was conveyed to visitors at the entrance of the park by means of a sign.

**TABLE 1:** Overview of the four different conditions.

Control condition	Visitors were not exposed to arguments or a question.
Arguments condition	Visitors were exposed to three different signs, each presenting a different argument for why not to lure the monkeys (see Figure 1).
Question condition	Visitors were exposed to one sign with the question “Why should you not lure the monkeys?” (see Figure 2).
Arguments and question condition	Visitors were first exposed to three signs with each presenting a different argument for why not to lure the monkeys, after which they were exposed to the sign with the question why they should not lure the monkeys.

### Study 1a: Rule Compliance

#### Procedure and Observation Protocol

**FIGURE 3:**
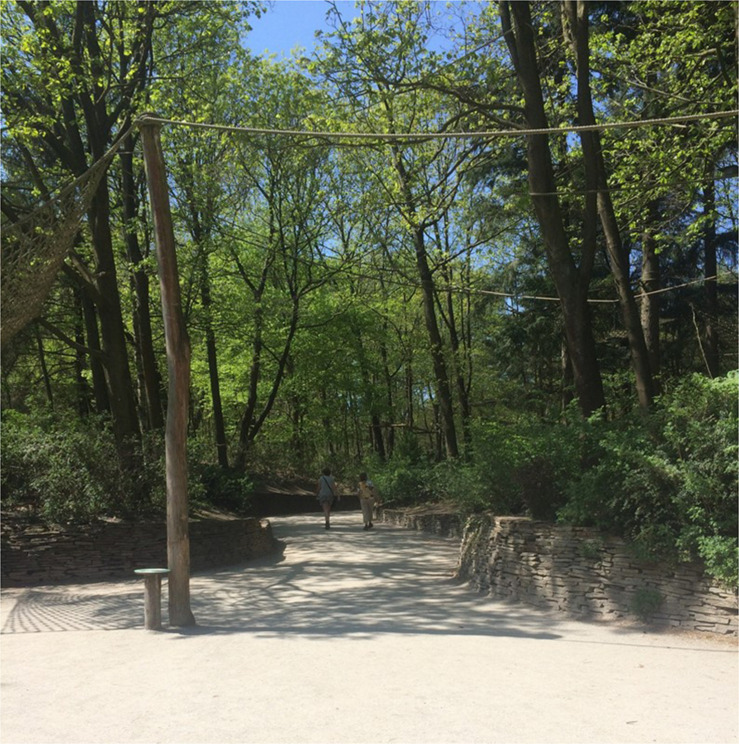
Observation area.

To observe visitors rule compliance behavior, we selected an area of 4 by 15 m in the middle of the free-ranging area (see Figure 3). Specifically, this was an area which all visitors would certainly walk through, where there were many possibilities for visitors to lure the monkeys, and that enabled unobtrusive observations of any rule violations.

One research assistant counted the total number of visitors that entered the area during the intervention period. In addition, three independent observers who were dressed as visitors discretely observed from a distance whether a visitor violated the rule, following a strict protocol (see Supplementary Information). Luring was operationalized as “purposefully trying to get a monkeys attention with the goal to lead them to a visitors desired location.” We decided to use three observers to ensure we could oversee the entire area. However, this made it possible that different observers recorded the same rule violator. Therefore, we divided the measurement area into three equal, consecutive sub-areas, with each observer tracking only the visitors that entered “their” sub-area. This means that observers could observe the same rule violator, but only once per area^3^.

Furthermore, in order to minimize the influence of external conditions, the observations within each condition were conducted at mornings that were similar in terms of visitor counts (i.e., medium-high) and weather conditions (i.e., sunny and around 19°C). In addition, because the behavior of the monkeys toward the visitors can differ within certain monkey family groups and within certain time periods (such as a difference in interaction with the visitors), the same family groups of monkeys were present in the free-ranging areas during the measuring periods of our study^4^.

To ensure that there were actually monkeys present at the location during all the measurement periods so that rule violations could be observed, the monkey caretakers put food in the observation areas 5 min before every half hour measuring period, which attracted the monkeys. In addition, every 15 min observers made a note of whether there were monkeys present in and around the measuring areas. We only included observation periods in which monkeys were present in the observation area (14 out of 16 observation periods; 87.5%).

#### Participants

All visitors of the Apenheul who entered the designated measurement area within the free-ranging area of the squirrel monkeys at the time of the study were included as participants (*N* = 2,264; 385 in the argument condition, 628 in the question condition, 371 in the combined condition and 880 in the control condition). Because participants needed to be able to read and understand the information on the signs, children shorter than 1.50 m (the average length of an 11-year-old in Netherlands during the time of this study lays between 1.43 and 1.48 m) were not included as participants. We assumed that all children older than 11 would be able to read and understand the text on the signs and used length as a proxy of age^5^.

### Study 1b: Perceived Importance of the Rule.

#### Procedure

At the terrace of a café situated next to the exit of the free-ranging area, visitors leaving the free-ranging monkey area were approached and asked whether they were willing to participate in the study. They were told that the purpose of the questionnaire was to understand visitor’s opinions and thoughts about the rules in the park. Visitors who agreed to fill out the questionnaire were explicitly asked to do so independently.

#### Questionnaire Measures

Participants were first asked to indicate how important they find the non-luring rule. Specifically, participants were asked to rate on scale from 1 (very unimportant) to 7 (very important) how important they thought the non-luring rule is, and how important they thought it is that visitors adhere to this rule. The two items formed a reliable scale (*r* = 0.75, *M* = 5.88, *SD* = 1.07).

Next, all participants were asked to indicate on a scale from 1 (very unpersuasive) to 7 (very persuasive) how persuasive they found each of the three arguments used on the signs. In addition, participants in all experimental conditions except the control condition indicated whether they had seen and read the signs. Participants who were provided with a question also indicated whether they had answered the question on the sign (yes/no).

Subsequently, participants who saw the sign with the question were asked what arguments they provided in favor of the non-luring rule, how they answered the question and indicated on a scale from 1 (very difficult) to 7 (very easy) how hard they found it to come up with these arguments. Results showed that participants in the question and the arguments and question combination condition found it relatively easy to come up with arguments, as mean scores were above the midpoint of the scale. Participants who were provided with arguments before they were provided with the question did not find it easier to answer the question (*M* = 4.91, *SD* = 1.60) than participants who were provided with the question alone (*M* = 5.30, *SD* = 1.36) [*t*(76) = 1.036, *p* = 0.303].

For all participants, the questionnaire concluded with questions on background information [gender, age, and seasonal card holder (yes/no)]^6^. Additionally, respondents indicated whether they would address other visitors if they saw them breaking the rule (yes/no). These results are not reported here as they are not relevant for the aim of this study. The complete questionnaire is available upon request.

#### Questionnaire Participants

In total 358 visitors filled out our questionnaire. Eight responses were not included in the final dataset because we had reason to believe participants did not fill out the questionnaire correctly or seriously (e.g., inconsistent answering^7^). Accordingly, our final dataset consists of 350 visitors of which 40% were men, 59% woman and 1% who did not indicate their gender. Participants were between 12 and 79 years old (*M* = 36.09; *SD* = 14.62).

### Data Analysis

In Study 1a we tested the differences in luring behavior between conditions with the z-proportion test (two-sided) for comparing proportions between different conditions. In Study 1b we conducted a two-way analysis of variance (ANOVA) to test the main and interaction effects of providing arguments (yes/no) and providing the question (yes/no) on the perceived importance of the non-luring rule. Next, we conducted planned contrast analyses to test the hypothesized differences between specific conditions. In order to correct for an increase of possible type-I error resulting from multiple comparison analysis while maintaining enough statistical power to prevent an increase in type-II error we applied a Tukey HSD test.

## Results

### Rule Compliance

**FIGURE 4:**
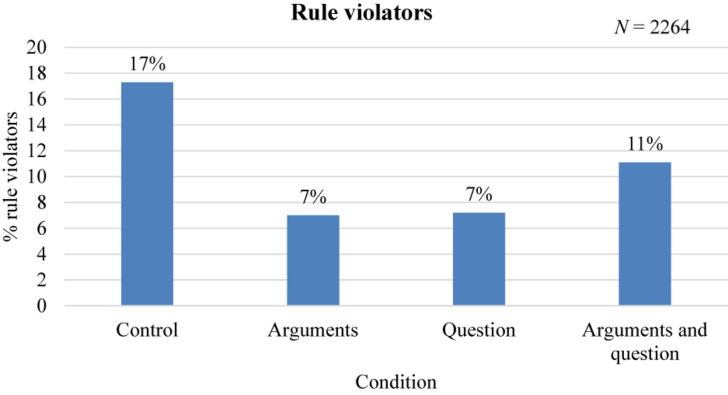
Percentage of observed visitors that broke the non-luring rule per condition.

Figure 4 shows that in support of Hypothesis 1a, a significant smaller percentage of visitors lured the monkeys when arguments in favor of the non-luring rule were given (7%), than in the control condition (17%) (*z* = 4.82, *p* < 0.001, *n* = 1265, 95% CI = 0.067, 0.138). In addition, as expected, visitors lured the monkeys significantly less when they were provided with the question alone (7%; *z* = 4.82, *p* < 0.001, *n* = 1508, 95% CI = 0.069, 0.133) and when both the arguments and the question were provided (11%) compared to the control condition (17%; *z* = 2.78, *p* < 0.01, *n* = 1251, 95% CI = 0.022, 0.103).

Interestingly, we found no support for Hypothesis 2a, as no significant differences were found between the argument and the question condition (*z* = 0.09, *p* = 0.928, *n* = 1013, 95% CI = 0.034, 0.031). Interestingly, the percentage of visitors that lured the monkeys when visitors were provided with both arguments and the question (11%) was *higher* compared to when only arguments were provided (*z* = 1.94, *p* = 0.052, *n* = 756, 95% CI = 0, 0.081) and when only the question was provided (*z* = 2.12, *p* < 0.05, *n* = 999, 95% CI = 0.001, 0.077; both 7%).

### Perceived Importance of the Rule

Table 2 shows the number of participants, and the mean and standard deviation for perceived importance of the non-luring rule per condition. An ANOVA showed a significant main effect of presenting the question, *F*(1, 345) = 6.48, *p* < 0.05, η*^2^_*p*_* = 0.018: visitors who were provided with the question (*M* = 6.03, *SD* = 0.94) perceived the rule to be more important than visitors who were not provided with the question (*M* = 5.73, *SD* = 1.18). No significant main effect was found for providing arguments *F*(1, 345) = 0.69, *p* = 0.408, η*^2^_*p*_* = 0.002, indicating that visitors who saw the sign with the arguments (*M* = 5.94, *SD* = 1.00) either alone or before seeing the sign with the question did not find the non-luring rule significantly more important than visitors that did not see the sign with the arguments (*M* = 5.83, *SD* = 1.15). The interaction effect was also not significant *F*(1, 345) = 1.23, *p* = 0.268, η*^2^_*p*_* = 0.004, indicating that the effect of providing the question did not differ depending on whether arguments were provided or not.

**TABLE 2:** Number of participants, means and standard deviations on the perceived importance of the rule per condition.

	***N***	**Mean**	**SD**
Control	92	5.63^a^	1.23
Arguments	81	5.85^ab^	1.11
Question	84	6.05^b^	1.01
Arguments and question	93	6.02^ab^	0.88

*Mean with unequal superscript differ significantly at *p* < 0.05.*

Next, planned contrast analyses showed that there was no significant difference on perceived importance between visitors in the argument condition and visitors in the control condition [*F*(1, 345) = 1.86, *p* = 0.523, 95% CI = −0.641, 0.198]. Yet, visitors in the question condition [*F*(1, 345) = 6.71, *p* = 0.047, 95% CI = −0.834, −0.001] did perceive the non-luring rule to be significantly more important than visitors in the control condition. Visitors in the combined condition perceived the rule to be somewhat more important than visitors in the control condition, but this difference was not statistically significant [*F*(1, 345) = 6.06, *p* = 0.068, 95% CI = −0.790, 0.019]. Even though visitors in the question condition found the non-luring rule slightly more important than visitors in the argument condition, this difference was not statistically significant either [*F*(1, 345) = 1.39, *p* = 0.640, 95% CI = −0.626, 0.233]. Furthermore, we found no significant difference in perceived importance of the rule between the combined condition and the arguments condition [*F*(1, 345) = 1.03, *p* = 0.741, 95% CI = −0.582, 0.254] or the question condition [*F*(1, 345) = 0.04, *p* = 0.997, 95% CI = −0.383, 0.447].

## Discussion

Rules are often implemented to instigate socially desirable behavior. It is assumed that arguments explaining the rationale behind the rule can convince people of the importance of the behavior and make individuals more likely to perform the behavior. We tested this assumption and further tested whether asking people themselves to generate arguments to engage in a socially desirable behavior, instead of providing these arguments to them by an external source, may be an even more effective way to promote rule compliance. When doing so, people convince themselves of the importance of the behavior (i.e., self-persuasion) instead of being convinced by an external source, which can make them more likely to follow the rule as they are motivated to be consistent with their own arguments. Furthermore, we investigated whether combining the two approaches, so that arguments are provided to people before they are asked to come up with their own arguments, would weaken the self-persuasion effect because people may not feel they themselves were the ones coming up with arguments, or whether it would strengthen the self-persuasion effect by making it easier for people to come up with arguments themselves.

As expected, we found that rule compliance was higher when arguments in favor of the rule were presented compared to when only the rule was communicated (i.e., control group), supporting Hypothesis 1a. Moreover, when individuals were asked to generate arguments in favor of the rule themselves, rule compliance was higher compared to the control condition, supporting our reasoning that asking individuals to generate arguments themselves can be effective in promoting rule compliance. Importantly, these findings show that subtle and simple persuasion techniques can be powerful in increasing rule compliance. Specifically, providing either three arguments, or using a sign to ask people to generate arguments for a socially desirable behavior, can result in a significant increase in rule compliance compared to a control condition. Particularly the latter finding is interesting, as it suggests that simple self-persuasion techniques – just asking a question on why a rule is important – can already be powerful. In addition, this finding suggests that intensive self-persuasion techniques, as used in most previous studies such as asking people to write down arguments or convince others using self-generated arguments (e.g., Stone et al., 1994; Baldwin et al., 2013; Arieli et al., 2014), might not be necessary to promote socially desirable behaviors.

Yet, we found that providing arguments and asking a question were about equally effective in promoting rule compliance, which does not support Hypothesis 2a that asking a question is more effective in stimulating rule compliance than providing arguments. One explanation for why presenting a question was not more effective than presenting arguments could be that we used a very relevant and credible source (i.e., de Apenheul, who is a clear expert in monkey well-being), which could be as persuasive as people generating arguments themselves. Arguments provided by a less credible and relevant source may have been less effective than arguments generated by an individual themselves (Kaufman et al., 1999; Ziegler et al., 2005; Smith et al., 2013). Further research is needed to test whether the expertise and credibility of the source may indeed affect the effect of arguments on rule compliance, and whether arguments provided by a less relevant and credible external source would have been less effective than self-generated arguments. Nonetheless, the finding that providing arguments and asking a question are both similarly effective in stimulating rule compliance could have important practical implications. Notably, our results suggest that providing arguments can be effective in situations in which people find it hard or might not be able to come up with arguments and hence are less able to persuade themselves, for example when it is a new topic where people know little about. In these situations, persuasive arguments can be used to stimulate rule compliance instead of asking a question.

Interestingly we found that presenting arguments and the question together did promote rule compliance but was less effective than presenting either arguments or a question alone. This implies that combining both approaches decreases rather than increases the effect of each intervention alone on rule compliance. Providing arguments before asking a question could weaken the positive effect of self-persuasion because people do not feel they themselves were the ones coming up with the arguments. It could be that providing arguments and asking a question about these arguments afterward, made visitors feel like they were being checked or tested if they read and understood the signs correctly. As a result, they may have reacted less positively to the signs than they did when they were only provided with arguments or a question. This may explain why rule compliance was not higher, and even lower, when presenting arguments and the question together compared to presenting either arguments or a question alone. However, future research is needed to test this reasoning. Further, we observed that visitors did not find it easier to come up with their own arguments when they were provided with arguments before asking the question. This may also explain why rule compliance was not higher (and even lower) in the combined intervention to each intervention alone; apparently, visitors did not find it difficult to come up with their own arguments in the first place. It is possible that providing arguments before the self-persuasion process is more effective in stimulating rule compliance than only asking the question when people find it hard to come up with arguments themselves; future research is needed to test this.

We found a different pattern of results for the perceived importance of the rule than for rule compliance. Specifically, we found that even though all visitors generally perceived the rule to be important, only visitors who were asked to generate arguments themselves perceived the rule to be even more important compared to the control condition. This supports hypothesis 2b and indicates that asking people to generate arguments in favor of a rule themselves makes it more likely that they convince themselves of the importance of this rule. The finding that both providing arguments and asking a question increased rule compliance but the perceived importance of the rule only increased when people came up with arguments themselves, suggests that there might be a different process underlying the effect of providing arguments and asking a question on increasing rule compliance. It may be that asking a question strengthens peoples’ intrinsic motivation act in line with the rule because people convince themselves of the importance of the behavior and hence will adopt the behavior because they want to act in line with what they find important themselves. In contrast, arguments provided by an external source may particularly strengthen extrinsic motivation, since people adopt the behavior because a relevant and knowledgeable source says it is important and not because they embrace the behavior themselves. Future research is needed to test whether providing arguments indeed elicits a different motivational process than asking people to generate arguments themselves.

Interestingly, although our results indicated a possible increase in the perceived importance in the combined condition compared to the control condition, the combination of providing arguments and asking a question was less effective in eliciting rule compliance than only providing arguments or the question alone. This suggests that not only there might be two different processes underlying each of the interventions but also that when these interventions are combined, these processes do not complement each other but rather undermine each other’s effectiveness. Indeed, research shows that extrinsic motivation can undermine the effects of intrinsic motivators (e.g., Deci et al., 1994). This finding may be important for promoting sustained engagement in socially desirable behavior, because people that are intrinsically motivated to perform a certain behavior, are more likely to perform this behavior over and again, also when no external control is present (Self Determination Theory, Deci and Ryan, 1985), in other situations (Steg, 2016), and show related behaviors (i.e., positive spillover, e.g., Truelove et al., 2014). In contrast, when people are more extrinsically motivated it is likely that they will only perform the behavior when interventions or sanctions are in place (Bolderdijk et al., 2011). For organizations and institutions this would imply that people are more likely to perform the desired behavior in other situations and that less surveillance is needed to control the maintenance of the rule. Future research is needed to determine whether asking people to generate arguments in favor of performing a behavior themselves indeed increases intrinsic motivation to adopt socially desirable behaviors and whether this is more likely to result in long term changes in behavior compared to providing arguments.

The strength of our current study is that we observed actual behavior in a real-life setting which highly increased the external validity of our results. However, as is common for field studies, the study set-up was bound by practical limitations. For example, we cannot rule out the possibility that people who complied to the rule in the observation area did break the rule throughout the entire free-ranging area. In addition, we used multiple observers to tally the amount of rule violations, to ensure we could oversee the entire area. Therefore, it may be possible that one rule-violator was tallied more than once. Yet, we found the same pattern of results when we look at one area in which there was only one independent observer, which suggests that this did not affect our results in important ways. Furthermore, the questionnaire was conducted in a busy environment where other people were present, which means that participants may have been distracted and had the opportunity to discuss their answers even though they were explicitly asked not to. In addition to the benefits gained from conducting field studies, it is important to replicate the same phenomenon using different research design to overcome these mentioned practical issues and accommodate the internal validity of our results.

In conclusion this study showed that providing arguments explaining the rationale behind a rule, asking people to come up with arguments themselves and a combination of these two interventions, are effective ways of enhancing rule compliance. However, our results suggest that asking a question is more likely to increase the perceived importance of the rule compared to not asking a question, which may result in longer lasting effects. Interestingly, combining both approaches so that arguments are offered before the self-persuasion process seemed to be less effective in eliciting rule compliance than either providing arguments or the question alone. These findings suggest that providing arguments in favor of a rule and asking people to come up with arguments themselves are both relatively easy and effective ways to increase rule compliance. Yet, when aiming to increase both rule compliance and the perceived importance of the rule, the most promising strategy appears to be to ask people to convince themselves to do the right thing.

## Data Availability Statement

The datasets generated for this study are not publicly available. However, access to the datasets can be requested to the corresponding author.

## Ethics Statement

The studies involving human participants were reviewed and approved by the Ethical committee of the psychology department of the University of Groningen. If applicable, verbal informed consent to participate in this study was provided by participants’ legal guardian/next of kin.

## Author Contributions

NL and KK originated the research ideas and designed the study. NL collected the data, performed the data analysis, and drafted the manuscript. KK, TB, and LS were engaged in several rounds of critical revisions of the manuscript. All authors approved of the final version of the manuscript to be published.

## Conflict of Interest

The authors declare that the research was conducted in the absence of any commercial or financial relationships that could be construed as a potential conflict of interest.

## Footnotes

1 The non-luring rule is conveyed to visitors by means of signs and an audio fragment at the main entrance of the park together with the other rules of the free-ranging area. Other rules include not feeding and petting the monkeys, no food consumption and packing all belongings in special “monkey-free bags.”

2 A pilot study (*N* = 27) was conducted to test the persuasiveness of these arguments. All three arguments were rated as persuasive; mean scores were above the midpoint of the 7-point scale ranging from 1 (not at all convincing) to 7 (very convincing).

3 Examination of the data of the middle area only (of which we are certain all visitors were only observed once) reveals a similar pattern of results as found for the entire observation area.

4 In order to ensure safe and appropriate interaction between the visitors and the monkeys, park stewards are present in all free-ranging area of Apenheul. To ensure that visitors did not feel observed and fear enforcements of the rule, stewards were instructed to keep an appropriate distance and refrained from acting when a visitor broke the non-luring rule.

5 Due to presence of large numbers of people in the area we observed, it was practically impossible to register demographics such as age and gender. Moreover, informing participants about the study, which would be needed to gather such data, could have influenced their behavior. However, an indication of the distribution of these characteristics can be found in the method section of the questionnaire study. Since participants in both studies came from the same population of visitors, we expect similar distributions.

6 There were no significant differences in participants’ age [*F*(3, 342) = 0.51, *p* = 0.628] and gender [*F*(3, 339) = 0.58, *p* = 0.678] between the four conditions, suggesting that the samples of visitors in each of the conditions were similar.

7 Inspection of the questionnaires revealed that a substantial number of participants corrected themselves while answering the items on the importance scale, suggesting that the anchors of the scale (very unimportant – very important) were initially misinterpreted by participants. Therefore, we checked all questionnaires for potential inconsistencies between answers on the different items of the perceived importance scale and between the perceived importance scale and other questions assessing the importance of the rule, such as willingness to address other visitors who lure the monkeys and the open-ended questions. All questionnaires that showed major inconsistencies (e.g., indicating the rule to be very unimportant and explaining why the rule is so important at the open-ended question or the other way around) were excluded from the dataset.
