# Supplementary material for: Convince Yourself to Do the Right Thing: The Effects of Provided Versus Self-Generated Arguments on Rule Compliance and Perceived Importance of Socially Desirable Behavior
Source: Front Psychol. 2020 Dec 23;11:613418. doi: 10.3389/fpsyg.2020.613418 (PMC7786400; doi:10.3389/fpsyg.2020.613418)
Supplement: Supplementary file 1 [file Data_Sheet_1.PDF]

Supplementary information

Luring protocol

A strict protocol was followed to decide which behaviors were considered luring and which were not. The protocol was developed in collaboration with park stewards and monkey caretakers, in accordance with the park's guidelines. Luring was operationalized as 'purposefully trying to get a monkeys attention with the goal to lead them to a visitors desired location'. The following behaviors were considered luring:

- The use of objects to gain attention from the monkeys, such as rustling bags or tapping twigs on the ground.
- Offering or providing the monkeys with objects such as twigs, pebbles or leaves with the intention to have them approach you.
- Sticking out the arm and or hand in the direction of the monkeys, paired with movements of the fingers or hands, or the making of sound in order to gain the attention of the monkeys.
- Sticking out the arm and or hand in the direction of the monkeys, offering or providing them a place to jump to or sit on.

Standing still or sitting down in an area where the monkeys roam around in the hope that they will (voluntarily) approach, is not considered luring. Additionally, making sounds or talking out loud to the monkeys is not considered luring if this is not paired with a physical act as described above, because the monkeys do not notice and are not disturbed by this.
